# The Effect of Sports Game Intervention on Children’s Fundamental Motor Skills: A Systematic Review and Meta-Analysis

**DOI:** 10.3390/children11020254

**Published:** 2024-02-16

**Authors:** Shengchuan Sun, Changzhou Chen

**Affiliations:** School of Physical Education, Shanghai University of Sport, Shanghai 200438, China; sunshengchuan@sus.edu.cn

**Keywords:** sports game, fundamental motor skills, children, intervention experiment

## Abstract

The purpose of the present study was to carry out a systematic review and a meta-analysis determining the effects of sports game intervention on fundamental motor skills in children. This paper presented a systematic review from 2001 to 2020, including the databases of Web of Science, EBSCO, Science, PubMed and Springer. (1) Participants comprised 1701 children age 3–12 years; (2) sports game interventions were investigated; (3) only studies using a Test of Gross Motor Skills assessment were included; (4) RCT were chosen as the study design to assess the impact of sports game interventions on children’s motor skills; (5) only culture-based sports game studies in the English and Chinese language were included. Two researchers independently reviewed and assessed the eligibility criteria. Out of the initial 1826 references, 12 studies with a total of 1608 participants were included in the meta-analysis. All interventions were implemented in preschool (kindergarten) or primary school populations, and all studies followed a randomized controlled trial design. The results demonstrated significantly superior development of fundamental motor skills in the intervention groups compared to the control groups (standardized mean difference = 0.30, *p* < 0.0001). The methodological quality of the included studies ranged from fair to excellent, and no evidence of publication bias was observed. Among them, the 12-week sports game intervention of 35 min each time three to four times a week had a better intervention effect, promoting children’s physical health and fundamental motor skills.

## 1. Introduction

Fundamental motor skills (FMS), sports, and health are integral components of curriculum offerings at various educational levels, from elementary to higher education. FMS, defined as organized basic movements involving two or more body segments [1], play a crucial role in supporting the overall development of body posture from childhood to adulthood. This encompasses locomotor, stability, and manipulative skills [2]. The elementary school curriculum prioritizes three core FMS—locomotor, stability, and manipulative skills—with the aim of fostering children’s engagement in recreational physical activities. The integration of these skills holds particular significance.

The primary objective of enhancing FMS is to establish a cohesive continuum that includes both fine and gross motor skills. These encompass locomotor skills (e.g., walking, running, jumping), object control skills (e.g., catching, throwing, kicking), and body control skills (e.g., balance, swinging). Fine and gross motor skills coexist harmoniously, contributing to students’ active participation in various physical activities, laying the foundation for their further physical development [3].

Physical education in primary schools extends beyond skill acquisition and activity participation, holding deeper significance. It provides students with opportunities to develop cooperation, teamwork, and leadership skills, influencing their personal growth throughout life. Additionally, physical education sparks an interest in health and fitness, fostering awareness of lifelong physical motor expression—a key aspect in addressing health issues among children and adolescents. The integration of fundamental motor skills within sports game interventions not only imparts comprehensive motor abilities but also profoundly influences cognitive and emotional development.

In elementary education, ‘Sports games’ denote purposefully tailored physical activities meticulously designed to foster fundamental motor skills in children. Customized to align with the developmental stage of young learners, these activities cultivate an enjoyable and engaging environment, thereby facilitating the acquisition and refinement of essential motor skills. Participation in sports games exerts a positive impact on children’s mood, a pivotal aspect in elementary school where engaging in games with peers is a primary activity. Notably, sports games contribute significantly to the enhancement of physical, fine, and gross motor skills, concurrently fortifying social skills, amplifying emotional intelligence, and refining personality. Moreover, engaging in sports games serves as a catalyst for bolstering mental health by encouraging active movement activities and fostering improvements in communication skills.

Numerous systematic reviews and meta-analyses have underscored the favorable influence of sports games on the enhancement of motor skills in children [4,5,6]. These activities, characterized by their inherent appeal, active engagement, and adaptability across subjects, serve as a catalyst for fostering interest in learning. This systematic review is meticulously designed to discern the nuanced attributes of motor skill interventions employing sports games and ascertain their efficacy in advancing fundamental motor skills. Leveraging a network meta-analysis, we systematically scrutinize the impact of diverse sports game interventions on the foundational motor skills of children, incorporating insights from recent studies. Consequently, this present systematic review aspires to furnish the academic community with the most contemporaneous information on this subject matter. The ultimate goal is to furnish the academic community with contemporary insights and a nuanced comprehension of the impact of sports game interventions on fundamental motor skills in children. Through synthesizing and critically analyzing recent research findings, this systematic review aligns with the broader context of academic discourse on the positive influence of sports games on motor skills in children. In doing so, it seeks to provide valuable insights for both researchers and practitioners engaged in enhancing children’s motor skills development.

## 2. Methods

The implementation process and report writing of this systematic review strictly comply with PRISMA (Preferred Reporting Items for Systematic Reviews and Meta-Analyses) guidelines.

### 2.1. Information Sources and Search Strategies

A systematic review of the literature was conducted before 28 February 2022 in the following five databases: Web of Science, EBSCO, Science Direct, PubMed, and Springer. Keywords included four groups: (1) Population: Preschool* OR Kindergarten OR early child* OR young child*; (2) Fundamental motor skills: motor OR movement OR fundamental AND skill OR ability OR competence OR performance OR proficiency; (3) Sports games: sports games OR sports game OR physical games OR PE games OR physical game OR sport play OR physical education games OR Sport recreation; (4) Intervention: intervene* OR effect* OR influence OR impact. These specified criteria were employed to ensure a comprehensive and rigorous selection process for the inclusion of relevant studies in the analysis.

### 2.2. Eligibility Criteria

All identified articles were evaluated by two reviewers (SSC and CCZ) for eligibility. Articles that met the following requirements were included in this review: (1) The subjects were preschool children, with an average age from 3 to 6 years old; (2) children should be given sports game intervention for at least 4 weeks; (3) the experimental design was a Randomized Controlled Trial (RCT); (4) fundamental motor skills were evaluated as dependent variables in each reviewed study.

The following criteria were applied to exclude ineligible articles in the future: (1) population: studies must focus on elementary school children; (2) exposure: interventions should center on sports games; (3) comparator: not applicable, as the primary focus will be on sports games; (4) outcome: research must report on the impact of sports games on fundamental motor skills; (5) study design: articles reporting quantitative results in detail, including journal articles, academic dissertations, and conference papers, will be included.

By applying these precise eligibility criteria, the systematic review aims to provide valuable insights into the effects of sports games on fundamental motor skills development in elementary school children.

### 2.3. Data Extraction and Processing

Two reviewers (SCS and CZC) independently extracted the relevant information using a standardized template. When data were missing or could not be extracted due to insufficient statistical report, the author(s) were contacted to request the missing data.

The procedure of extracting content and coding was as follows. First, we captured the basic details of each study, including the names and nationalities of authors, and the year of publication. Second, we collected and processed the basic details of the subjects, including sample size, age, and education level. Third, we captured data on the following five exercise prescription variables: frequency, intensity, duration, type, and intervention period.

Exercise frequency was classified according to the number of exercise sessions per week, as follows: low frequency: ≤2 times; moderate frequency: 3–4 times; high frequency: ≥5 times. Exercise intensity was classified into three levels as low, moderate, and vigorous. Exercise duration (the minutes each session lasted) was classified as follows: short: ≤45 min; moderate: >45 min to ≤60 min; long: >60 min. Intervention period was classified according to the length of the intervention period, as follows: short: 4–12 weeks; mid-length: 13–24 weeks; long: >24 weeks.

### 2.4. Assessment of Study Quality

To evaluate the quality of the literature collection, the authors utilized the Cochrane Risk of Bias Tool for Randomized Trials (ROB2). This widely recognized tool enables a comprehensive evaluation of potential biases present in the included studies. The risk of bias assessment was performed by SCS and CZC to ensure consistency and accuracy. In instances where differing views arose among the assessors, these discrepancies were resolved through rigorous discussion and consensus building. By employing this robust approach, the assessment of study quality aimed to ensure the credibility and reliability of the systematic review’s findings.

## 3. Result

**Figure 1:**
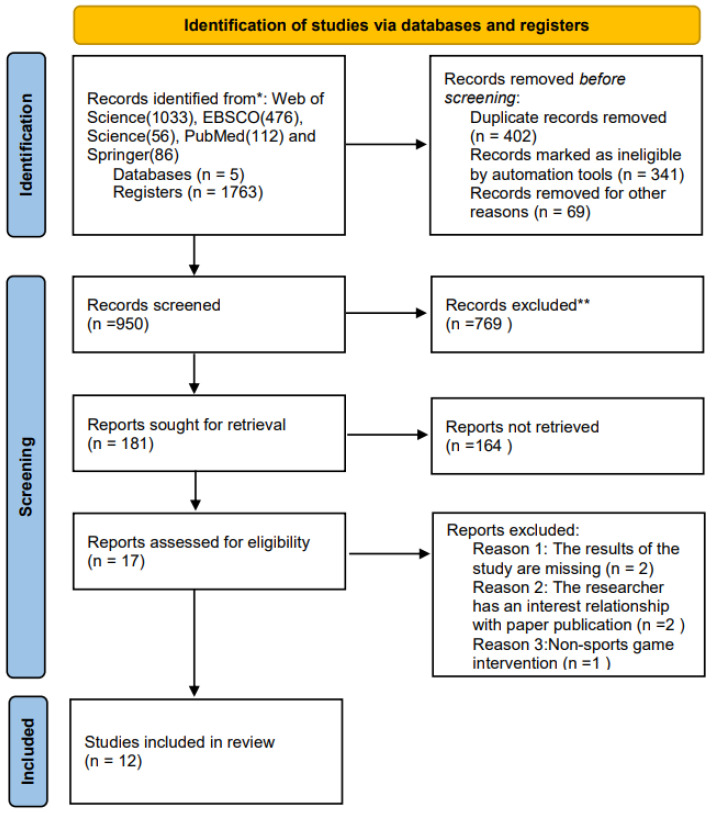
Literature selection flow diagram.*: In this study, a search for relevant literature was conducted across multiple scholarly databases, including Web of Science, EBSCO, Science, PubMed, and Springer. Specifically, 1033 records were identified from Web of Science, 476 from EBSCO, 56 from Science, 112 from PubMed, and 86 from Springer, resulting in a total of 1763 records. **: During the data processing phase, an automated approach was implemented to exclude 326 records, supplemented by a manual curation process that led to the exclusion of an additional 262 records.

The information depicted in Figure 1 can be derived. The number of articles in 2001–2006, 2007–2014, and 2015–2020 accounted for 25%, 50%, and 25% of the total included literature, respectively. Studies from North America, South America, and Europe accounted for 33%, 8%, and 42% of the included literature, respectively. The United States accounted for 33%. Greece accounted for 17%. New Zealand, Brazil, and Finland accounted for 17%, 8%, and 8%, respectively. Except for China, no authors from other Asian countries have published studies that meet our inclusion criteria.

**Figure 2:**
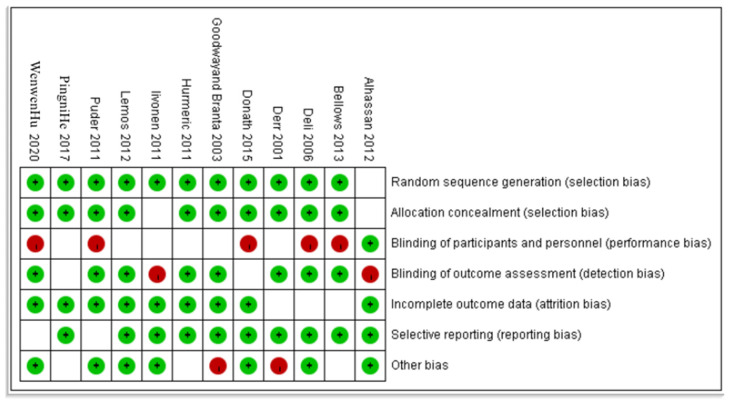
Document quality evaluation chart.

As shown in Figure 2, among them, there was 1 article with a score of more than six, 6 articles with a score of more than five, 1 article with a score of less than five, and 12 articles all achieved a low risk of bias.

As shown in Table 1, a total of 1826 literature studies were retrieved, and 1582 were included after screening. Two references were supplemented according to the included literature and related reviews, and 12 were finally determined. Table 1 shows 12 articles that were included from 2001 to 2020. The sample size was from 15 to 342 students, for which seven articles used TGMD measurement tools. Two article used PDMS and APM tools; two articles used the National Physical Fitness Standard (Children’s Part).

### 3.1. Effect of Intervention Duration on Fundamental Motor Skills Development

In terms of of single intervention time, 50% of the studies focused on 30–40 min [1,6,8,9,12,15]. Secondly, 42% of the studies were conducted within 41 to 50 min [10,11,13,14,16], and <30 min [7] accounted for 8%. 

### 3.2. Intervention Outcomes and Their Effectiveness

FMS was the most frequently-measured outcome, which was confirmed by several studies, suggesting it could be improved by sports game interventions [17,18]. According to the results of meta-analysis, an immediate and moderate effect of training (three to four times a week for more than 35 min each time for more than 12 weeks) was found on motor performance, indicating sports game interventions are effective for improving FMS in children. 

Other than motor performance, more than a half of the included studies assessed physical condition, emotional, and psychological factors. Overall, sports game interventions had a moderate and immediate effect on these outcomes in children based on the meta-analysis. However, it is worth noting that cognitive functions were the targeted outcomes in most studies (70%). Additionally, children’s self-concept or self-esteem regarding motor competence and physical competence were the most common intervention outcomes. 

Although previous studies have showed that sports game interventions have been remarkably successful in improving FMS as well as cognitive, emotional, and psychological factors in youths [19], challenges still remain to improve the sustainability of these training effects. This study found that the significant effect of training on this outcome occurred only immediately after the intervention, but was not sustained in the short term (4–8 weeks). Therefore, future studies should strive to optimize interventions and improve the sustainability of the effects of training on children’s basic motor skills.

Apart from FMS, cognitive, emotional, and psychological perspectives, the positive intervention effects on FMS were fully supported by the 12 studies according to the qualitative results. Meanwhile, the impact of physical play intervention on basic motor skill intervention and satisfaction with physical activity and participation in youths was considered inconclusive due to inconsistent experimental results. In a recent report [20], an ecological model was used to summarize the effects of physical play interventions on the improvement of basic motor skills in youths. 

Specifically, it emphasized the importance of participation in sports games and the relationship between participation and basic motor skill learning. In addition, a large amount of evidence shows that youths’ participation in sports games can develop better fundamental motor skills, especially in leisure time sports activities. Therefore, it is suggested that we use sports game activity participation as a variable in future motor skill interventions.

The sensitivity analysis (unadjusted weight) of the effect of sports game intervention on children’s short-distance running performance was significantly improved when only studies with an average age of 3–6 years old and RCT studieswere included.

Compared with the control group, the scores of basic motor skills in the intervention group were significantly higher than those in the control group. When the examination changed over time, the basic motor skills score of the intervention group increased significantly. However, the basic motor skill score of the control group did not change. We examined the control group and the intervention group, respectively, when examining the impact of gender throughout the study. For the control group, we observed no interaction between time and gender (*p* = 0.49), nor time (*p* = 0.49) or gender (*p* = 0.13). However, a significant interaction between time and gender was observed in the intervention group (*p* = 0.002). The main effect showed that male children scored significantly higher than female children in basic motor skills (*p* = 0.04).

The preliminary descriptive statistics in Table 1 highlight the descriptive statistics and average scores of participants at baseline. During the whole period, significant intervention effects were observed, including object control ability (*p* < 0.001) and gross motor ability (*p* < 0.001), and the improvement of the FMS total score was the most obvious (*p* < 0.001). Post hoc analysis showed that the intervention group had significant changes in the total goal control (*p* = 0.002, *d* = 0.35) and total athletic performance (*p* < 0.0001, *d* = 0.17) between baseline examination and post-intervention, and between baseline examination and retention examination (goal control *p* < 0.0001, *d* = 1.31, exercise *d* = 0.75). It was observed that the total FMS intervention effect of the intervention group was significantly improved compared with the control group.

### 3.3. Test Situation Effect of Testing Assessment on FMS

TGMD-2 [1,6,8,9,10,11,12] was first used in physical education in the United States, and has been widely used worldwide because of its cross-cultural adaptability and operability of measuring and evaluating indicators. Pdms-2 [7] and APM [13] accounted for 4%, respectively. on the evaluation and treatment of exercise status of children with disabilities aged 0-6 years; because of its complicated testing process, there is no direct evidence to prove that it can be used in a unified manner across regional and cross-cultural differences. 

### 3.4. Intervention Results

After evaluation, the post-intervention scores in the intervention group exhibited a significant increase compared to both the pretest and the control group. This observed improvement was evident in numerous studies [1,6,7,8,9,10,11,12,14,15,16], constituting 92% of the cases. Conversely, in 8% of the studies, as indicated by one study [13], there was no notable difference in post-test scores between the intervention group and either the pretest or the control group, suggesting the ineffectiveness of the intervention.

## 4. Discussion

The primary objective of this systematic review was to discern the distinctive characteristics of motor skill interventions tailored for children through sports games and assess their effectiveness in fostering fundamental motor skills. The investigation revealed variations in intervention characteristics concerning participant recruitment, setting measurements, and the types of sports games employed. Notably, motor performance, cognitive, emotional, and psychological factors emerged as the most prevalent outcome categories. Employing meta-analytic methods, immediate and moderate training effects were validated for the aforementioned outcome categories. Furthermore, the frequency of intervention and the dose (length of training in minutes) proved to be significant factors influencing the magnitude of these effects. This outcome aligns with a meta-analysis conducted by Clemente et al. [17], reporting an overall moderate effect of interventions (weighted Cohen’s *d* = 0.56). These findings substantiate the recommendations of the Child Motor Behavior Society for effective interventions, underscoring the importance of tailored sports game interventions for children.

From the literature reviewed, there was no significant difference in the test results and effect sizes between the INT and CON trials. We found that contrary to the physiological considerations of the dose–response principle, a longer intervention period produced a more pronounced skill improvement effect, and a shorter intervention period showed a smaller effect. A single type of sports game intervention makes the FMS intervention process more tedious. Over time, it is more likely to cause children and experimenters to lose interest, and may lead to a loss of compliance and enthusiasm of relevant personnel. In addition, the adaptability of the intervention program still has insufficient intervention depth, and the intervention content needs to be continuously adjusted over time to match the appropriate stimulation depth.

The current meta-analysis also evaluated the effects of sports game prescription on the improvement of children’s fundamental motor skills. The results of this study showed that the types of sports game, the duration of intervention, and the frequency of intervention are potential moderators of this relationship.

Although there are obvious gender differences in the effects of the intervention on fundamental motor skills [20,21,22], which may be related to the differences in physical activities between boys and girls and daily life behaviors and habits [23], it is generally beneficial and enhances the motor skills of both genders. The fundamental motor skills of girls (balance) and boys (kicking) did not respond equally to the effects of FMS intervention.

Only a few studies have specifically addressed gender differences. These studies summarize the clear implications of FMS interventions (one study shows that boys improve better [24], two studies show that girls improve better [25,26]). Some studies have shown that boys have better object control skills than girls [11,12,13,27]. However, there were no significant gender differences in motor abilities [11,12,28]. For instance, there was a lack of evidence for gender differences in motor skills related to actual ability and perceptual ability among the children tested. For games with controlled objects, perhaps boys may just get more encouragement, reinforcement, and stimulation. 

Boys may benefit more from interventions targeting object control skills. Four studies [11,12,13,29] have shown that sports game intervention has a more lasting impact on boys, producing a more obvious effect on the improvement of boys’ basic motor skills. However, another three studies [11,28,29] showed that there was no difference in the improvement of motor skills between boys and girls. Although some preliminary studies on FMS have shown that boys experience a greater effect than girls in FMS intervention [3,30], this cannot be concluded based solely on these few publications on gender differences. Among them, previous experiments [31] and subsequent experiments [32] have confirmed that sports game intervention plays a positive role in improving children’s object control ability [33,34]. Only a few studies have confirmed the different effects of sports game intervention on boys and girls, so we cannot conclude that girls or boys benefit more from sports game intervention in terms of FMS. Therefore, whether girls or boys truly benefit more from FMS intervention will need to be established by future publications using experimental data to support their conclusions. Therefore, future research should consider the universality or gender specificity of intervention methods, the acceptability and effectiveness of different methods, and the consequences of positive and negative interventions.

The development of FMS is constantly evolving, as it adapts to predictable changes in the lives of growing children, and its development is affected by various factors closely related to their growth and development. The development of FMS is essential for children’s physical, social, and psychological development. However, children’s FMS does not develop naturally with age. Mastering FMS requires education, exploration, encouragement, and mutual feedback. According to the summary of this study, children’s early motor skill proficiency is positively correlated with the frequency of participation in sports games. In addition, the motor performance of children with motor developmental defects observed in early childhood is still obvious in adolescence. Therefore, early childhood is a particularly important period for the development of motor skills. Studies have confirmed that the development and improvement of the brain plays an important role in the development of children’s basic motor skills and the completion of various motor tasks [34]. Through the research and summary of the included literature, it is found that young girls have strong body movement ability, while young boys have strong object control ability. In addition, the difference in interest between different genders shows that boys prefer ball games and thus develop a higher level of object control ability, which may also be the reason for the difference in maintenance effect between boys and girls after intervention. Improving the development of practical motor skills as well as perceptual motor ability in youths can provide more opportunities for the development of various perceptual, social, and cognitive skills.

### 4.1. Moderators of Training Effects

The results showed that sports game intervention frequency and duration were significant moderating factors. This suggests that these two factors play a greater role in regulating the size of the training effect. This review also demonstrated a positive relationship between the intervention dose (total minutes) and the therapeutic effect on motor performance. Specifically, longer treatment durations were associated with higher efficacy in enhancing motor skills among the participants. We also found that regardless of other intervention components (e.g., duration of each training session, total reps), a higher frequency of practice in sports game programs, such as 4–5 reps/week, significantly improved the effect of training on athletic performance. Specifically, treatment lasting at least 9 weeks significantly improved FMS performance in youths. All of these findings support the importance of the amount of experience in motor skill learning, as younger children tend to take longer to complete the learning process. Our review provided researchers and participants with empirical evidence of training doses for future interventions. At the same time, it is important to note that the optimal training dose to produce long-term training effects needs to be determined in future studies.

It should be noted that interventions such as those we performed in a school setting, for children with poor exercise ability, using usual care as a control condition, involving interventions lasting ≥9 weeks, and designed with a high-quality approach, tend to be more effective in improving youths’ exercise ability. Implementing motor skill interventions in schools have several advantages, including maximizing the sustainability of the interventions and minimizing the costs associated with implementation. Because the purpose of an intervention is to achieve meaningful engagement in all aspects of life, the involvement of parents and other important people (e.g., teachers, coaches) is very important to maximize the potential training effects. Future research is therefore needed to fully understand the role of significant others in promoting short- and long-term motor and mental performance in youth.

Though the present review has numerous strengths such as including studies with an RCT design, examining the short-team and long-term intervention effects, and determining the moderators of training effects, it has its limitations. Firstly, the pooled effect sizes were not weighted on the risk of the bias level of studies. Secondly, the retention effects on motor performance as well as cognitive, emotional, and psychological factors were based on a relatively small number of studies and were quite likely to be influenced by publication bias. Thirdly, the current review was limited to the search terms and current databases used, and was restricted to peer-reviewed journal articles published in English since 2001. Lastly, this systematic review and meta-analysis was not registered in any international registration system.

This paper is limited by language constraints, as only studies published in English were included in the retrieval process. The potential risk of bias in the methodological quality of the included literature, can influence our assessments of effectiveness and results of the interventions. As the fundamental motor skills assessment tools of the included literature are often different, they make horizontal comparisons challenging and inconvenient. As the included studies did not adequately address differences in motor patterns, comparisons could not be made, which limited its wider applicability.

### 4.2. Strengths and Limitations

Above all, the included studies represented six different countries and regions; therefore, the samples could represent children from different cultures and backgrounds. The findings of this review might be generalized to a larger population. In spite of these strengths, our review has some limitations. First, according to the study intervention characteristics, the sports game intervention duration or frequencies were not consistent. In addition, limited information regarding sports game frequencies or intensity were presented. Second, although this review only retrieved RCTs using TGMD as the sole instrument in assessing FMS, different versions of this instrument (PDMS-2, APM, and TGMD-2) might lead to different results. That is, the data accuracy and interpretation might have been compromised across the four versions. Some studies offered a variety of sports games, whereas others only employed certain predetermined sports games, which limited the generalization of specific sports game used in FMS interventions.

This systematic review describes and quantitatively summarizes the beneficial effects of sports game intervention on the development of children’s FMS. Sports game intervention can effectively improve children’s athletic ability and FMS; however, this only works over the short term. The intervention effects on physical fitness are also supported by the qualitative summary. Further, the effects of motor skill interventions are more robust in interventions that utilize a larger training dose and a high frequency of practice. Future basic motor skill interventions need to first determine the sustainability of training effectiveness, and then test the impact of motor skill interventions on promoting children’s activities and participation. Future basic motor skill interventions need to first determine the sustainability of the effectiveness of sports game interventions, and then test the impact of motor skill interventions on promoting children’s activities and participation.

## 5. Conclusions

This systematic review comprehensively examines and quantitatively synthesizes the positive effects of interventions involving sports games on the development of children’s fundamental motor skills (FMS). While acknowledging the effectiveness of these interventions in improving both athletic ability and FMS in children, it is important to emphasize that these effects are primarily observed in the short term. The qualitative summary further reinforces the beneficial influence of sports game interventions on physical fitness.

Furthermore, the study demonstrates that interventions targeting motor skills are more effective when they involve longer training sessions and a higher frequency of practice. These findings have implications that go beyond the current discussion, suggesting potential avenues for future research in the field of basic motor skill interventions.

Moving forward, it is crucial to carefully examine the sustainability of the effectiveness of motor skills training in order to advance interventions for basic motor skills. Future research should prioritize the evaluation of the long-term impact of sports game interventions on fundamental motor skills. Additionally, there is a pressing need to explore the broader impact of motor skill interventions on promoting children’s engagement in physical activities and overall participation.

In summary, this systematic review not only enhances our understanding of the short-term benefits of sports game interventions on children’s FMS, but also serves as a catalyst for future research directions. The pursuit of sustainability in training effectiveness and the multifaceted impact of motor skill interventions on children’s activities and participation are important areas that require in-depth exploration in subsequent investigations.

## Figures and Tables

**Table 1:** Intervention characteristics of the included literature.

Study	Country	Participants	PA Measurement & Method	Intervention Duration & Measurement Period	Measuring Tool	Intervention Results
Alhassan, 2012 [6]	USA	INT = 114 CON = 114	Intervention group: structured sports game intervention Control group: free play	24 weeks	TGMD-2	Intervention group > Control group
Bellows, 2013 [7]	USA	INT = 98 CON = 103	Intervention group: structured sports game intervention Control group: routine sports activities	18 weeks	PDMS-2	Intervention group > Control group
Deli, 2006 [8]	Greece	INT = 28 CON = 27	Intervention group: structured sports game intervention Control group: free play	10 weeks	TGMD	Intervention group > Control group
Derr, 2001 [9]	Greece	INT = 35 CON = 33	Intervention group: structured music game intervention Control group: free game activities	70 weeks	TGMD	Intervention group > Control group
Donath, 2015 [1]	New Zealand	INT = 22 CON = 19	Intervention group: ball control games Control group: daily sports activities	6 weeks	TGMD-2	Intervention group > Control group
Cotrim, 2012 [10]	Brazil	INT = 15 CON = 15	Intervention group: group cooperative sports games Control group: free play	45 weeks	TGMD-2	Intervention group > Control group
Goodway and Branta, 2003 [11]	USA	INT = 70 CON = 74	Intervention group: structured sports game intervention Control group: free play	12 weeks	TGMD	Intervention group > Control group
Hurmeric, 2011 [12]	USA	INT A = 22 INTB = 25 CON = 25	Intervention Group A: Object Control Sports Game Intervention Intervention group B: addition of action skills courses guided by professional teachers on the basis of group A Control group: routine sports activities	8 weeks	TGMD-2	Intervention group A ≈ Intervention group B > Experimental group
Iivonen, 2011 [13]	Finland	INT = 38 CON = 40	Intervention group: structured sports game intervention Experimental group: unstructured physical education curriculum	8 weeks	APM	Intervention group > Control group
Puder, 2011 [14]	New Zealand	INT = 313 CON = 313	Intervention group: sports game intervention at home, school, gymnasium Experimental group: regular physical education courses	36 weeks	Shuttle, run (20 m), obstacle course, balance beam and platform. multidimensional sports style	Intervention group > Control group
HuWenwen, 2020 [15]	China	INT = 68 CON = 60	Intervention Group: Functional Sports Game Intervention Control group: ordinary sports game intervention	12 weeks	‘National physique measurement standard (children)’	Intervention group > Control group
He Pingni, 2017 [16]	China	INT = 15 CON = 15	Intervention Group: Functional Sports Game Intervention Control group: free play	15 weeks	‘National physique measurement standard (children)’	Intervention group > Control group

TGMD: Test of Gross Motor Development, PDMS-2: Peabody Development Motor Scale—2nd Edition, APM: Allekouluikäistenlasten Psyko Motorisettaidot.

## Data Availability

Due to privacy and ethical considerations, the raw data supporting the findings of this study cannot be publicly deposited or shared. However, a de-identified subset of the data or summary statistics may be made available upon reasonable request and after obtaining necessary ethical approvals. Researchers interested in accessing the data are encouraged to contact the corresponding author “chenchangzhou@sus.edu.cn” for further guidance.
